# Does Branched-Chain Amino Acids Supplementation Modulate Skeletal Muscle Remodeling through Inflammation Modulation? Possible Mechanisms of Action

**DOI:** 10.1155/2012/136937

**Published:** 2012-02-14

**Authors:** Humberto Nicastro, Claudia Ribeiro da Luz, Daniela Fojo Seixas Chaves, Luiz Roberto Grassmann Bechara, Vanessa Azevedo Voltarelli, Marcelo Macedo Rogero, Antonio Herbert Lancha

**Affiliations:** ^1^Laboratory of Applied Nutrition and Metabolism, School of Physical Education and Sports, University of São Paulo, P.O. Box 05508-030 São Paulo, SP, Brazil; ^2^Laboratory of Molecular and Cellular Physiology of Exercise, School of Physical Education and Sports, University of São Paulo, P.O. Box 05508-030, São Paulo, SP, Brazil; ^3^Department of Nutrition, School of Public Health, University of São Paulo, P.O. Box 01246-904, São Paulo, SP, Brazil

## Abstract

Skeletal muscle protein turnover is modulated by intracellular signaling pathways involved in protein synthesis, degradation, and inflammation. The proinflammatory status of muscle cells, observed in pathological conditions such as cancer, aging, and sepsis, can directly modulate protein translation initiation and muscle proteolysis, contributing to negative protein turnover. In this context, branched-chain amino acids (BCAAs), especially leucine, have been described as a strong nutritional stimulus able to enhance protein translation initiation and attenuate proteolysis. Furthermore, under inflammatory conditions, BCAA can be transaminated to glutamate in order to increase glutamine synthesis, which is a substrate highly consumed by inflammatory cells such as macrophages. The present paper describes the role of inflammation on muscle remodeling and the possible metabolic and cellular effects of BCAA supplementation in the modulation of inflammatory status of skeletal muscle and the consequences on protein synthesis and degradation.

## 1. Introduction

 Skeletal muscle presents unique features that allow it to respond to several exogenous stimuli. This characteristic is named “plasticity” [1]. Exercise and nutrition are examples of such stimuli that may promote adaptive responses in skeletal muscle in terms of structure and function [2–4]. For example, there are some reports describing that mechanical stimuli, particularly resistance exercise, may induce histological changes such as fiber type transition and profile and increase in cross-sectional area [5], and alterations in muscle function [6, 7]. Branched-chain amino acids (BCAA), especially leucine, are also well-known nutrients that may influence the adaptive response of skeletal muscle. Leucine supplementation has been described as a potential nonpharmacological tool able to stimulate both muscle anabolism and decrease catabolism [8, 9] and to modulate glucose homeostasis [10]. Furthermore, leucine can act synergistically with exercise to improve the efficiency and effectiveness of these adaptive responses [11].

 Currently, there are some cellular pathways that partially explain why BCAA supplementation may promote such responses in skeletal muscle. Most of these consistent evidences were observed on incubated cells, which have contributed to elucidate important mechanisms regarding amino acids modulation of skeletal muscle protein turnover. However, we have to consider that such conditions are considerably different from the human body. Although experimental animals (rodents) represent an *in vivo* model, it may also present distinct results when compared to humans. Recently, our group observed that due to differences in muscle metabolism, rodents may respond differently from humans to amino acids supplementation [12]. Although the same signaling pathways are found in rodent and human cells the response of these models to amino acids supplementation present individualities that may compromise the extrapolation of results.

 The mammalian target of rapamycin (mTOR) pathway is a signal-dependent cascade that responds to a variety of stimuli ranging from growth factors and mitogens to amino acid deprivation and hypoxic stress. It has been well characterized that mTOR pathway has a pivotal role in modulating protein translation initiation through eukaryotic initiation factors (eIFs) and kinases, which in turn alter the phosphorylation status and activity of several proteins in this cellular pathway [13]. Amino acids supplementation is involved in signaling to upstream proteins, responsible for sensing and triggering (mTOR, human vacuolar protein sorting 34 (hVps4), calcium-related proteins) [14], as well as downstream proteins, responsible for ribosome initiation complex formation (eIF4E, eIF4E-binding protein 1 (4E-BP1), eIF4F complex) [15, 16]. Additionally, it has been shown that BCAA can also interact with the proteolytic machinery (ubiquitin proteasome system—UPS) in order to attenuate muscle wasting [17]. This response may partially involve the protein kinase Akt/PKB, which also participates in glucose homeostasis and muscle hypertrophy. Regarding the proteolytic machinery, Akt/PKB is known to phosphorylate the transcription factor forkhead box class-O (FoxO), which translates the two majority genes (or E3 ligases) of muscle atrophy: atrogin-1 and muscle RING-finger protein-1 (MuRF-1) [12, 18, 19], to phosphorylate mTOR and stimulate protein synthesis, and to modulate glucose transporter 4 (GLUT4) to the sarcolemma [20]. In view of this, these cellular pathways (synthesis and degradation) are not distinct and may be controlled by amino acids through indirect genomic and nongenomic actions.

 Although much attention has been given to the role of amino acids in these pathways, the responsiveness of skeletal muscle to these nutrients may be limited. For instance, amino acids infusion stimulate muscle protein accretion until it reaches a *plateau *[21]. This condition, known as “anabolic resistance to amino acids”—the inability of skeletal muscle to maintain or increase its protein mass by appropriate nutritional stimulation [22] occurs because skeletal muscle protein synthesis is refractory to hyperaminoacidemia. Thus, it appears that the optimal action of amino acids on skeletal muscle growth occur in combination with other exogenous stimuli (e.g., exercise) or in situations characterized by disruption of organic homeostasis (e.g., cancer, diabetes, muscle disuse, sepsis, chronic heart failure). In this context, the inflammatory status has a considerable role and the innate immune system (responsible for cytokines and chemokines production) should be carefully considered.

 The focus of this paper is to discuss the possible metabolic and cellular roles of BCAA supplementation on the inflammatory status of skeletal muscle and the effects on protein synthesis and degradation. It is possible that, in some conditions, the administration of these amino acids could exert an anti-inflammatory role or indirectly modulate the inflammatory status and balance of the system and/or the muscle cell in order to favor the biological response and tissue adaptation.

## 2. The Role of Inflammation in Skeletal Muscle

**Figure 1:**
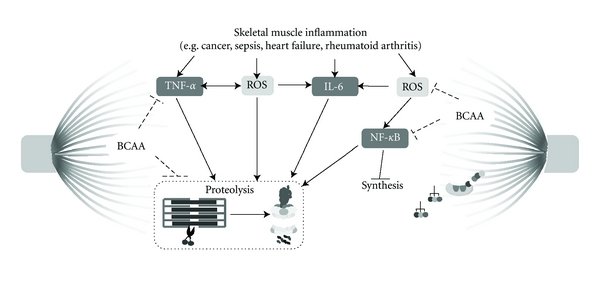
Signaling pathways linking skeletal muscle inflammation and remodeling Possible role of BCAA action. Skeletal muscle inflammation can increase the expression and activity of factors such as TNF-*α*B, ROS, IL-6, and NF-*κ*B which in turn could contribute to protein degradation and attenuate protein synthesis. On the other hand, BCAA supplementation could counteract such effects by suppressing skeletal muscle proteolysis and stimulation protein synthesis. BCAA: branched-chain amino acids; IL-6: interleukin 6: NF-*κ*B, nuclear factor kappa B: ROS: reactive oxygen species; TNF-*α*: tumor necrosis alpha.

The healing of injured muscle is composed of sequential but overlapped phases of injury, inflammation, regeneration, and fibrosis. Injury and inflammation predominate the first few days after injury, followed by regeneration. When there is a severe injury the muscle does not recover completely and forms fibrotic tissue approximately two weeks after injury [23] (Figure 1).

The inflammatory response is an important phase of the natural healing process. During this phase there is a release of several types of cytokines and growth factors to increase the permeability of blood vessels and chemotaxis of inflammatory cells, such as neutrophils and macrophages. These cells contribute to the degradation of damaged muscle tissue by releasing reactive oxygen species (ROS) and producing proinflammatory cytokines [24–27] such as tumor necrosis factor alpha (TNF-*α*), interleukin-1 (IL-1), and IL-6 that regulate the inflammatory process [28, 29]. The role of these cells is quite complex and they can promote both injury and repair. A detailed discussion of their action is beyond the scope of this paper and has been reviewed elsewhere [30].

### 2.1. Tumor Necrosis Factor Alpha (TNF-*α*)

Some systemic inflammatory cytokines such as TNF-*α* and IL-1*β* have direct catabolic effects on skeletal muscle. The cytokine TNF-*α* plays a key role in the skeletal muscle wasting present in chronic diseases, such as cancer, sepsis, and rheumatoid arthritis, conditions in which a raise in the plasma TNF-*α* concentration have been described [27, 31]. TNF-*α* impairs muscle protein synthesis [32, 33] by destabilizing myogenic differentiation and altering transcriptional activity [34] and increases muscle loss [35, 36] by targeting proteins to the ubiquitin-proteasome- mediated degradation pathway [37–39]. TNF-*α* has also an effect on satellite cells and muscle regeneration. In vitro studies have shown that exposure of myoblasts to TNF-*α* inhibits their differentiation [40, 41]. The release of ROS induced by TNF-*α* induces degradation of the inhibitor-*κ*B (I*κ*B), which allows the nuclear factor-kappaB (NF-*κ*B) to translocate to the nucleus and to activate the transcription of several *κ*B-dependent genes such as those encoding proinflammatory cytokines, and breakdown of MyoD and myogenin (regulators of the transition from proliferation to differentiation) in the proteasome [34].

Although most research has focused on the muscle wasting effects of TNF-*α*, under specific conditions this cytokine can also promote muscle protein synthesis and stimulate satellite cell proliferation and differentiation [38, 42]. Among the factors that mediate the different effects of TNF-*α* on protein synthesis or degradation are the state of cell differentiation and the concentration of TNF-*α*. Chen et al. [42] have shown that the effects of TNF-*α* on myogenesis and muscle regeneration are concentration dependent: a low concentration of TNF-*α* (0.05 ng/mL) promoted the differentiation of cultured myoblasts while higher concentrations (0.5 and 5 ng/mL) inhibited it. Furthermore, differences in the expression of the TNF-*α* receptor on the surface of different cell types may explain the variable effects of this cytokine. In primary myotubes, low doses of TNF-*α* (1 ng/mL) stimulated maximal protein synthesis, while a much higher dose (50 ng/mL) was required to stimulate maximal protein synthesis in C2C12 myotubes [43]. Therefore, the effects of TNF-*α* depend on the concentration and exposure duration: low concentrations help the repair process, while high and prolonged exposure impairs the regeneration process. It is possible that other factors, such as insulin growth factor 1 (IGF-1) and inflammatory cytokines mediate the effects of TNF-*α* on protein synthesis/degradation, but their roles are still unclear [43, 44].

Elevated levels of TNF-*α* have also been implicated in sarcopenia, the age-related loss of muscle mass, strength, and function [45]. It is a key factor contributing to loss of functional mobility, frailty, and mortality in the elderly [46, 47]. Inflammation, which generally increases with age, is a key factor contributing to sarcopenia, and high level of TNF-*α* is partly responsible for the decrease in muscle protein synthesis that occurs in the elderly [48–51]. Greiwe et al. [52] have found elevated levels of TNF-*α* mRNA and protein in the skeletal muscle of elderly (81 ± 1 years) when compared to young (23 ± 1 years) men and women. The same authors also showed that resistance exercise decreased TNF-*α* expression in the elderly group, suggesting that TNF-*α* contributes to the age-related muscle wasting, and that resistance exercise may attenuate this process by suppressing TNF-*α* expression.

### 2.2. Lipopolysaccharides (LPS)

LPS are components of the outer wall of Gram-negative bacteria. It is well known that Gram-negative infection (or the administration of lipopolysaccharides) causes loss of skeletal muscle protein. The decrease in muscle mass results from increases in the rate of proteolysis and decreased rates of protein synthesis [53, 54]. A decrease in mTOR activity may explain, at least in part, the impaired muscle protein synthesis. Frost et al. [55] have shown that a combination of LPS and IFN-gamma dramatically downregulated the autophosphorylation of mTOR and its substrates S6K1 and 4E-BP1 via an increased expression of iNOS (NOS2) and excessive production of nitric oxide (NO).

### 2.3. Interleukin-6 (IL-6)

Studies in mice have shown that overexpression of IL-6 may increase muscle atrophy [56]. However, under certain conditions IL-6 can also promote muscle growth. In vitro data has shown that recombinant IL-6 stimulates myoblasts differentiation [57]. Furthermore, Al-Shanti et al. [58] demonstrated that IL-6 in combination with TNF-*α* stimulate growth of myoblasts. Therefore, the role of IL-6 in regulating muscle mass appears to be concentration dependent: when overexpressed it may stimulate muscle atrophy, whereas its insufficiency inhibits muscle growth.

### 2.4. Nuclear Factor Kappa B (NF-*κ*B)

The NF-*κ*B serves as a key responder to changes in the environment. It mainly controls the expression of genes involved in the immune response, but it also regulates the expression of genes outside the immune system and is therefore able to influence several aspects of normal and disease physiology [59, 60]. The classic form of NF-*κ*B, a heterodimer of the p50 and p65 subunits, is retained in the cytoplasm through interactions with I*κ*B inhibitory proteins. Inducing stimuli lead to the phosphorylation and degradation of I*κ*B by I*κ*B kinases (IKK), allowing NF-*κ*B to enter the nucleus and regulate gene expression [61]. Chronic activation of NF-*κ*B is related to skeletal muscle pathology. In mice, NF-*κ*B activation causes severe muscle wasting [62] and NF-*κ*B is a key factor in cytokine induced loss of skeletal muscle [63].

Exercise may activate several signaling cascades and increase the production of ROS, which activate NF-*κ*B [42, 64] in muscle [65, 66]. The exercise induced increase in NF-*κ*B induces acute-phase proteins and also proinflammatory genes that facilitate the regenerative response in damaged tissues. The involvement of NF-*κ*B in muscle damage has been shown in several reports where exhaustive exercise has caused increases in NF-*κ*B binding activity [67]. Roberts et al. [68] have also shown that the inflammatory response to exercise is attenuated by chronic training, demonstrating that the activity of NF-*κ*B can be seen as a beneficial mediator of exercise-induced adaptations to cellular stress. Furthermore, exercise training may induce local anti-inflammatory effects in skeletal muscle [69].

A training program can also exert an inhibitory effect on NF-*κ*B DNA binding [70, 71]. Regular physical training leads to several adaptations in the vascular, oxidative, and inflammatory systems, suggesting that transcriptional regulators of the various nitric oxide synthase (NOS) isoforms by NF-*κ*B play a key role in training-induced adaptations [72, 73]. Lima-Cabello et al. [74] have demonstrated that the effects observed after a bout of acute exercise on the NF-*κ*B signaling were attenuated by submaximal eccentric exercise training for 8 weeks. Also, the levels of nNOS, iNOS and eNOS expression and nitrotyrosine formation decreased when compared to the acute group.

## 3. The Possible Role of BCAA Supplementation on Muscle Inflammation

**Figure 2:**
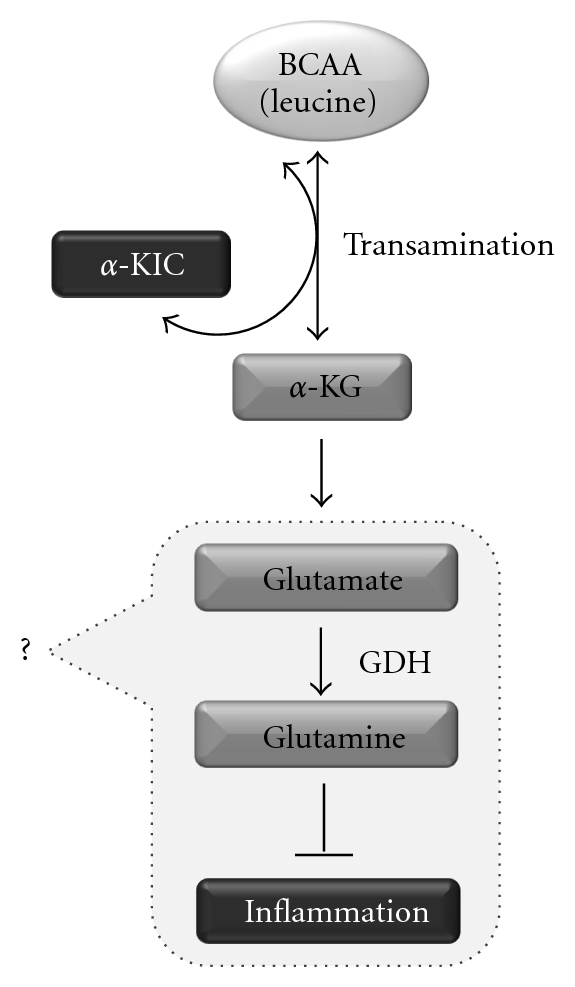
Possible role of glutamine as an intermediary of BCAA action on skeletal muscle inflammation. BCAA could modulate inflammatory response through glutamine synthesis. Such reaction occurs through BCAA transamination and generation of glutamate from *α*-KG. Glutamate is then converted to glutamine by glutamine synthetase. *α*-KG: alpha-ketoglutarate; *α*-KIC, *α*-ketoisocaproate; BCAA: branched-chain amino acids; GDH: glutamate dehydrogenase.

Unlike other amino acids, the most active enzyme system for BCAA transamination is found in the skeletal muscle rather than the liver [75]. The first reaction involved in the catabolism of BCAA is the reversible reaction of transamination by isoenzymes BCAT (branched-chain aminotransferase) found in both cytosol and mitochondria, which convert amino acids into their respective keto acids (branched-chain keto acids—BCKA), being the *α*-ketoisocaproic acid (KIC) for leucine, *α*-keto-*β*-methylvaleric acid (KMV) for isoleucine, and *α*-ketoisovaleric acid (KIV) for valine. The BCKAs formed may undergo oxidative decarboxylation reactions and/or be released in the blood stream and taken up by different tissues, where they are resynthesized to BCAA or oxidized [75]. It is well known that the amino group from BCAA can be incorporated into the *α*-keto-glutarate (*α*-KG) producing glutamate through glutamate dehydrogenase (GDH). The glutamate can lose the amino group for oxalacetate (OAA) through glutamate-oxalacetate aminotransferase (GOT) producing aspartate to be used in the purine cycle for regeneration of adenosine monophosphate (AMP) from inosinic acid. Glutamate can also be metabolized by glutamine synthetase producing glutamine through ATP-dependent incorporation of NH^+^_3  _[76] (Figure 2).

Muscle cells present very high concentrations of glutamate and a low activity of GDH [77]. Therefore, the amino group released from BCAA could be easily reincorporated by BCKA (reamination) to produce BCAA or directed to the liver to be oxidized. Alanine can also be synthetized from BCAA since the glutamate-pyruvate aminotransferase can produce pyruvate which can be transaminated to alanine through pyruvate aminotransferase [78, 79]. However, this reaction appears to occur only in situations characterized by absence of energy (i.e., fasting) since skeletal muscle also presents high concentrations of alanine [75].

The intracellular pool of amino acids can be derived from biosynthesis (i.e., nonessential amino acids) or from transfer across the plasma membrane (i.e., essential amino acids). Transfer across biological membranes can occur through active (Na^2+^-dependent) or passive transport (Na^2+^-independent) due to their ionic nature [80]. In some instances the process of transfer involves not only the entry but exit of amino acids (exchange). The transport system of glutamine is called System A (Na^2+^-dependent) and the one of leucine is called System l (L; Na^2+^-independent), which are integrated. The glutamine entry in the cell requires Na^2+^, while leucine entry requires exit of glutamine. In conditions where the requirement of glutamine is increased (i.e., catabolic illness), theoretically the intracellular content of glutamine is decreased [81]. The decrement in intracellular pool of glutamine may impair leucine transport to inside the cell. On the other hand, the increase of glutamine transport outside the cell may favor the entry of leucine into the cell. If leucine transport is stimulated, the final result of catabolism could increase the availability of glutamine to the cell through glutamate. However, there are no studies evaluating the effects of leucine and glutamine supplementation under inflammatory conditions.

BCAA can indirectly modulate the inflammatory status of muscle cells through glutamine production but this reaction appears to occur only in situations characterized by high glutamine consumption and/or decreased glutamate concentrations (i.e., catabolic illnesses, cancer, burning, and sepsis).

Glutamine is an amino acid that plays an important role in maintaining cell function. Ehrensvard et al. [82] first described the importance of glutamine to survival and proliferation of cells such as kidney, intestine, liver, specific neurons in the central nervous system (CNS), pancreatic *β* cells, and cells of the immune system. It is widely known that cells of the immune system such as lymphocytes, macrophages, and neutrophils use high rates of glutamine and many functional parameters of immune cells such as T-cell proliferation, B-lymphocyte differentiation, macrophage phagocytosis, antigen presentation, and cytokine production, plus neutrophil superoxide production and apoptosis are enhanced by glutamine. Under pathological conditions, which increase the activity of these cells glutamine is extremely used as substrate [83–85].

It has already been demonstrated that the availability of glutamine influences the production of cytokines such as interleukin- (IL-) 2 in cultured rodent lymphocytes [86] and, IL-2, IL-10, and interferon-*γ* (IFN-*γ*) in cultured human lymphocytes [87, 88]. Studies have also demonstrated that glutamine may play an important role on NF-*κ*B signal transduction pathways, contributing to the attenuation of local inflammation [89–92]. When inhibited in the cytoplasm NF-*κ*B is bound to an inhibitory protein: I*κ*B. In inflammatory conditions, the I*κ*Bs are phosphorylated by the action of specific protein kinases, such as the IKB kinase complex (IKK) at two serine residues with addition of ubiquitin by ubiquitin ligase and degraded by 26S proteasome complex resulting in liberation of NF-*κ*B. Activated NF-*κ*B then binds to the cognate DNA-binding sites inducing gene transcription that regulates the innate and adaptive immune response (i.e., T-cell development, maturation, and proliferation) [93–95].

Regarding skeletal muscle remodeling, NF-*κ*B acts as FoXO, a transcription factor of MuRF-1 gene which promotes sarcomeric degradation by UPS [62]. Several cytokines also have their gene expression modulated by NF-*κ*B (i.e., TNF-*α* and IL-1*β*). It was demonstrated that IL-1*β* presents significant correlation with skeletal muscle cross-sectional area and, therefore, can be considered as an atrophic modulator [96]. NF-*κ*B also promotes the transcription of the inducible isoform of nitric oxide synthase (iNOS) [97] which leads to insulin resistance through nitrosylation of the insulin receptor (IR) [98]. Under such conditions, the mTOR translation pathway has impaired signal transduction through proteins involved in translation initiation such as insulin receptor substrates (IRS), Akt, and 4E-BP1 [18]. Therefore, inflammation may modulate muscle remodeling through both synthetic and catabolic pathways.

Counteracting such effects, BCAA (especially leucine) has demonstrated to be a strong nutritional stimulus able to increase skeletal muscle protein synthesis and attenuate protein degradation. For example, Hamel et al. [99] demonstrated that leucine presents one of the strongest inhibitory effects upon UPS in muscle cells when compared to the other essential amino acids (for details about the antiproteolytic effects of leucine, please see Zanchi et al. [8] and Nicastro et al. [9]. Furthermore, it has already been demonstrated that BCAA can stimulate the phosphorylation of proteins involved in the mTOR pathway such as Akt, mTOR, 4E-BP1, eIFs, p70S6k in order to improve the protein turnover of the cell [100, 101]. Since BCAA do not present kinase nor phosphatase activity, their effects appear to be mediated by hVpS34 and calcium-related proteins [102].

Thus, BCAA can directly modulate the protein turnover of the muscle cell in order to counteract the catabolic and anti-anabolic effects of the inflammatory stimulus. Additionally, under pathological conditions, BCAA may influence the inflammatory status of the cell through glutamine production. However, this reaction appears to occur only in situations characterized by a high need of glutamine synthesis.

## 4. A Possible Link between Oxidative Stress and BCAA-Mediated Inflammatory Effects

Skeletal muscle cells continuously produce reactive oxygen species (ROS), which can be generated by various cell organelles and enzymes, such as mitochondria, NAD(P)H oxidases, xanthine oxidoreductases, and nitric oxide synthases, whereas their biological activity is opposed by an array of endogenous enzymatic and nonenzymatic antioxidants [103]. Normally ROS play important physiological roles in skeletal muscle homeostasis and function [104, 105]. However, a disturbance in the state of the well-balanced control of oxidant production and antioxidant activity, known as oxidative stress, in turn, is commonly observed during aging and is characteristic of several pathological conditions such as cancer, diabetes, muscle disuse, sepsis, and chronic heart failure [106]. It has been reported that this oxidative stress directs muscle cells into a catabolic state and that chronic exposure leads to wasting [107]. Oxidative damage may contribute to skeletal muscle dysfunction [108] and oxidants may stimulate expression and activity of skeletal muscle protein degradation pathways [38, 109].

There are several evidences showing that the generation of ROS is one mechanistic link between inflammation and skeletal muscle dysfunction and degradation. ROS produced by infiltrating immune cells may cause direct injury to muscle tissue or activate catabolic signaling. Alternatively, inflammatory cytokines can interact with muscle receptors to initiate catabolic signaling wherein ROS are key mediators of this response, acting as second messengers [107, 110, 111]. Accordingly, overexpression of TNF-*α* in transgenic mice [112] and single intraperitoneal doses of this cytokine [103] promotes muscle wasting that can be attenuated by antioxidants [103, 108]. On the other hand, ROS activate transcription factors (e.g., NF-*κ*B and AP-1) and upregulate expression of proinflammatory genes such as TNF-*α*, IL-6 and C-reactive protein, which are involved in the pathogenesis of inflammation [113, 114].

Although administration of BCAA has been investigated as a tool that could exert an anti-inflammatory role or indirectly modulate the inflammatory status in order to favor the biological response and tissue adaptation, less is known about the relationship between this strategy and oxidative stress modulating skeletal muscle structure and function. There are some emerging reports describing that ROS modulate the efficiency and effectiveness of the adaptive responses of skeletal muscle induced by some BCAA, especially leucine [115, 116]. Regarding BCAA supplementation and oxidative stress, an interesting study [117] has shown that this nonpharmacological strategy increases expression of genes involved in antioxidant defense and reduces ROS production in cardiac and skeletal muscles in middle-aged mice, which was accompanied by preserved skeletal muscle fiber size, enhanced physical endurance and increased average life span. Of interest, BCAA-mediated effects were even more remarkable in middle-aged mice submitted to long-term exercise training (running 30 to 60 min 5 days/week for 4 weeks). In young animals (4–6 months old), BCAA supplementation was ineffective.

Aging has been described as a condition characterized by anabolic resistance to nutrients, especially amino acids, which impairs muscle protein synthesis and contributes to muscle wasting. Such resistance is partially associated to oxidative stress and low-grade inflammation and may be attenuated by chronic anti-inflammatory treatment. Recently, Smith et al. [118] demonstrated that older adults who received omega-3 fatty acids for 8 weeks increased the hyperaminoacidemia-hyperinsulinemia-induced muscle protein synthesis when compared to the control group (corn oil), which was accompanied by greater phosphorylation of mTOR^Ser2448^  and p70S6k^Thr389^. Furthermore, the authors observed decreased serum levels of TNF-*α* in the omega-3-supplemented group. Therefore, the anti-inflammatory action of nutrients such as omega-3 may attenuate anabolic resistance in order to favor amino acid-induced muscle protein synthesis.

Concerning BCAA supplementation, Marzani et al. [115] demonstrated that old rats supplemented with leucine and with an antioxidant mixture (rutin, vitamin E, vitamin A, zinc, and selenium) showed higher protein synthesis when compared to old-control animals and that these effects could be mediated through a reduction in the inflammatory state, which decreased with antioxidant supplementation. Under inflammatory conditions, such as aging, anabolic resistance occurs mainly because of elevated proinflammatory cytokines. Thus, antioxidant supplementation may attenuate anabolic resistance and therefore favor leucine action on skeletal muscle protein turnover.

## 5. Conclusion and Perspectives

BCAAs present unique features in skeletal muscle protein metabolism. It is well accepted that their catabolic reactions can be easily modulated through alterations in metabolic demands, such as in inflammatory status. However, it is unknown if BCAA can directly modulate the status of proteins involved in inflammatory pathways and if this effect could reflect on protein turnover. Since glutamine is highly consumed by inflammatory cells, it appears to be a mediator of BCAA and inflammation but this reaction is dependent of glutamate content and GDH activity in skeletal muscle. Future studies should address the effects of BCAA, glutamine and the amino acids transporter activity under proinflammatory conditions.
